# Contemporary Thyroid Nodule Evaluation and Management

**DOI:** 10.1210/clinem/dgaa322

**Published:** 2020-06-03

**Authors:** Giorgio Grani, Marialuisa Sponziello, Valeria Pecce, Valeria Ramundo, Cosimo Durante

**Affiliations:** Department of Translational and Precision Medicine, Sapienza University of Rome, Rome, Italy

**Keywords:** biopsy, risk assessment, risk factors, TIRADS, ultrasonography, watchful waiting

## Abstract

**Context:**

Approximately 60% of adults harbor 1 or more thyroid nodules. The possibility of cancer is the overriding concern, but only about 5% prove to be malignant. The widespread use of diagnostic imaging and improved access to health care favor the discovery of small, subclinical nodules and small papillary cancers. Overdiagnosis and overtreatment is associated with potentially excessive costs and nonnegligible morbidity for patients.

**Evidence Acquisition:**

We conducted a PubMed search for the recent English-language articles dealing with thyroid nodule management.

**Evidence Synthesis:**

The initial assessment includes an evaluation of clinical risk factors and sonographic examination of the neck. Sonographic risk-stratification systems (e.g., Thyroid Imaging Reporting and Data Systems) can be used to estimate the risk of malignancy and the need for biopsy based on nodule features and size. When cytology findings are indeterminate, molecular analysis of the aspirate may obviate the need for diagnostic surgery. Many nodules will not require biopsy. These nodules and those that are cytologically benign can be managed with long-term follow-up alone. If malignancy is suspected, options include surgery (increasingly less extensive), active surveillance or, in selected cases, minimally invasive techniques.

**Conclusion:**

Thyroid nodule evaluation is no longer a 1-size-fits-all proposition. For most nodules, the likelihood of malignancy can be confidently estimated without resorting to cytology or molecular testing, and low-frequency surveillance is sufficient for most patients. When there are multiple options for diagnosis and/or treatment, they should be discussed with patients as frankly as possible to identify an approach that best meets their needs.

The prevalence of thyroid nodules in the general population is high—up to 60% as documented by high-resolution ultrasonography—but very few of these lesions ultimately prove to be malignant (about 5%) (1). Although epidemiological studies suggest a small but real increase in the incidence of thyroid cancer, likely stemming from exposure to environmental risk factors (2), the growing number of thyroid cancer diagnoses is largely attributable to the increasingly widespread use of diagnostic imaging technology and medical surveillance, together with improved access to health care in general, all of which favor the discovery of small, subclinical thyroid nodules and small papillary thyroid cancers. These considerations have raised concern over the costs and potential morbidity associated with the short- and long-term management of patients with thyroid nodules, which includes periodic outpatient visits and cervical ultrasound examinations, fine-needle aspiration biopsy (FNAB), genomic testing, and, in some indeterminate cases, diagnostic thyroid lobectomy. On the whole, there is a clearly perceived need for a more refined, tailored, and careful approach to the management of these highly prevalent lesions. Similar considerations can be extended to that amount of nodules that are proven to be malignant, but have a low-risk phenotype, and can be safely managed through more conservative surgery or even active surveillance programs (3, 4). The aim of this review is to provide an overview of currently recommended practices for the initial workup and subsequent management of patients with thyroid nodules.

## Search strategy

We conducted a search of PubMed using the following terms: “thyroid nodule”[tiab] AND (“disease management”[MeSH Terms] OR (“disease”[All Fields] AND “management”[All Fields]) OR “disease management”[All Fields]) AND ((“2017/01/01”[PDAT]: “2020/12/31”[PDAT]) AND English[lang]). From the 215 records returned, we selected the articles that were most relevant, with a preference for more recent publications. We scanned the reference lists of the papers identified to find other relevant articles.

## Primum non nocere

Clinicians encountering patients with thyroid nodules today are faced with the task of avoiding the overdiagnosis of low-risk cancers without jeopardizing the chances of identifying those rare advanced or higher risk tumors that will require prompt treatment. Overdiagnosis implies the discovery of conditions that will never cause morbidity or death. As such, their identification can trigger a cascade of deleterious events: people are transformed into patients, with inevitable emotional consequences and potential exposure to risks related to overmedicalization and overtreatment. At the public health level, overdiagnosis overstretches the capacities of health systems, increases spending, and subtracts resources from patients with the greatest health care needs. It is little wonder that the U.S. Preventive Services Task Force now recommends against thyroid cancer screening in asymptomatic adults, because its harmful effects outweigh its potential benefits (5). The recommendation against screening does not apply to patients with known risk factors for thyroid cancer (e.g., childhood radiation exposure in the form of radioactive fallout or radiotherapy, including low-dose forms for benign conditions; inherited syndromes associated with thyroid cancer; a family history of thyroid cancer), but even in these cases, the benefits of early detection have yet to be demonstrated (6). So, the implementation of sonographic screening programs for thyroid nodules is discouraged.

## Initial assessment

The initial assessment of a clinically evident or incidentally discovered thyroid nodule includes cervical sonography and evaluation of clinical risk factors. Depending on the results that emerge, the use of other diagnostic tools, such as FNAB for cytology and molecular testing, will be indicated for a small subset of the lesions.

## Thyroid ultrasound and sonographic risk-stratification systems

Thyroid ultrasonography (US) is the first-line tool for thyroid imaging. The thyroid gland is superficial in the neck, with the posterior border usually located less than 4 cm below the skin surface. High-resolution linear probes provide excellent image definition of the gland. The examination is safe and painless, requires no preparation, and can be performed rapidly in different care settings. To characterize thyroid nodules and obtain an initial estimate of their risk for malignancy, the examiner should focus on the echogenicity of the nodule; its composition (solid, cystic, mixed), shape, and margins; the presence within the nodule of calcifications or other hyperechoic foci; and the characteristics of all cervical lymph nodes (7). Findings consistently associated with malignancy include hypoechogenicity; infiltrative, irregular, or lobulated margins; intranodular microcalcifications; and a taller-than-wide shape. In addition to the nodule itself, all US studies must include a thorough exploration of all cervical lymph node compartments, and the presence of any suspicious lymph nodes should be noted (8). The diagnostic sensitivity and specificity of these features vary, and no single feature has proved capable of reliably distinguishing malignant lesions from those that are benign (9). In addition, recognition and reporting of these features are characterized by substantial interobserver and even intraobserver variability (10).

To address these shortcomings, several national and international professional organizations have developed US-based risk-stratification systems (often referred to as Thyroid Imaging Reporting and Data System or TIRADS, terms derived from those used for breast cancer imaging) that assign thyroid nodules to categories characterized by increasing risks (or risk ranges) for cancer, based on the presence or absence of the above-mentioned nodule features (Table 1) (11-15).

**Table 1.** An Overview of the Standardized Thyroid Nodule US Risk Stratification Systems Proposed or Endorsed by National or International Practice Guidelines^*a*^

Risk Score	AACE/AME/ACE (12)	ATA (13)	EU-TIRADS (14)	K-TIRADS (15)
Suspicious US features	▪ Marked hypoechogenicity ▪ Spiculated or lobulated margins ▪ Microcalcifications ▪ Taller-than-wide shape ▪ Extrathyroidal growth ▪ Pathologic adenopathy	▪ Irregular margins (infiltrative, microlobulated) ▪ Microcalcifications ▪ Taller-than-wide shape ▪ Rim calcifications with small extrusive soft-tissue component ▪ Evidence of extrathyroidal extension	▪ Non-oval shape ▪ Irregular margins ▪ Microcalcifications ▪ Marked hypoechogenicity	▪ Microcalcification ▪ Taller-than-wide shape ▪ Spiculated/microlobulated margins
Category	**Low-risk:** Risk of malignancy: 1% FNAB > 20 mm (selective)^*b*^ Cysts (fluid component >80%). Mostly cystic nodules with reverberating artifacts and not associated with suspicious US signs. Isoechoic spongiform nodules, either confluent or with regular halo.	**Benign:** Risk of malignancy: <1% FNAB is not indicated Purely cystic nodules (no solid component)	**Benign (EU-TIRADS 2):** Risk of malignancy: ≈ 0% FNAB is not indicated pure/anechoic cysts; entirely spongiform nodules	**Benign:** Risk of malignancy: <1-3 FNAB ≥ 20 mm Spongiform Partially cystic nodule with comet-tail artifact Pure cyst
		**Very low suspicion:** Risk of malignancy: < 3%FNAB ≥ 20 mm or observation Spongiform or partially cystic nodules without any of the US features defining low-, intermediate-, or high-suspicion patterns	**Low-risk (EU-TIRADS 3):** Risk of malignancy: 2%- 4%FNAB > 20 mm Oval shape, smooth margins, isoechoic or hyperechoic, without any feature of high risk	**Low suspicion:** Risk of malignancy: 3%-15%FNAB ≥15 mm Partially cystic or isohyperechoic nodule without any of 3 suspicious US features
		**Low suspicion:** Risk of malignancy: 5%-10%FNAB ≥ 15 mm Isoechoic or hyperechoic solid nodule, or partially cystic nodule with eccentric solid area without: microcalcifications, irregular margin, extrathyroidal extension, taller than wide shape		
	**Intermediate-risk:** Risk of malignancy: 5–15% FNAB >20 mm Slightly hypoechoic (vs. thyroid tissue) or isoechoic nodules, with ovoid-to-round shape, smooth or ill-defined margins May be present: Intranodular vascularization Elevated stiffness at elastography, Macro or continuous rim calcifications Indeterminate hyperechoic spots	**Intermediate suspicion:** Risk of malignancy: 10–20%FNAB ≥10 mm Hypoechoic solid nodule with smooth margins without: microcalcifications, extrathyroidal extension, or taller-than-wide shape	**Intermediate-Risk (EU-TIRADS 4):** Risk of malignancy: 6%-17% FNAB >15 mm Oval shape, smooth margins, mildly hypoechoic, without any feature of high risk	**Intermediate suspicion:** Risk of malignancy: 15%- 50% FNAB ≥10 mm Solid hypoechoic nodule without any s uspicious US feature or partially cystic or isohyperechoic nodule with any of the following: microcalcification, nonparallel orientation (taller-than- wide), spiculated/ microlobulated margin
	**High-risk:** Risk of malignancy: 50%-90%^*c*^ FNAB ≥10 mm (5 mm, selective)^*d*^ Nodules with ≥1 of the following: Marked hypoechogenicity (vs. prethyroid muscles) Spiculated or lobulated margins Microcalcifications Taller-than-wide shape (AP > TR) Extrathyroidal growth Pathologic adenopathy	**High suspicion:** Risk of malignancy: >70%-90% FNAB ≥10 mm Solid hypoechoic nodule or solid hypoechoic component of partially cystic nodule with ≥1 of the following: Irregular margins (infiltrative, microlobulated) Microcalcifications Taller-than-wide shape Rim calcifications with small extrusive soft tissue Extrathyroidal extension	**High-risk (EU-TIRADS 5):** Risk of malignancy: 26%-87% FNAB > 10 mm Nodules with ≥ 1 of the following: Non-oval shape Irregular margins Microcalcifications Marked hypoechogenicity	**High suspicion:** Risk of malignancy: > 60 FNAB ≥ 10 mm (>5 mm selective) Solid hypoechoic nodule with any of the following: Microcalcification Nonparallel orientation (taller-than-wide) Spiculated/ microlobulated margin

From Tumino D, Grani G, Di Stefano M, et al. Nodular thyroid disease in the era of precision medicine. *Front Endocrinol (Lausanne).* 2020;10:907.

*Abbreviations*: AACE/ACE/AME, American Association of Clinical Endocrinologists/American College of Endocrinology/Associazione Medici Endocrinologi; ATA, American Thyroid Association; EU-TIRADS, European Thyroid Association (ETA) Thyroid Imaging Reporting and Data System; K-TIRADS, Korean Society of Thyroid Radiology Thyroid Imaging Reporting and Data System.

^*a*^The TIRADS developed and endorsed by the American College of Radiology (ACR) is also widely used. Unlike the systems shown in the table, in which risk is defined by the association of 2 or more nodule features, the ACR system individually assesses 5 key aspects of the nodule (composition, echogenicity, shape, margins, and echogenic foci) and expresses the result in terms of a numerical score. The nodule is then assigned to a risk class based on the sum of the 5 scores.

^*b*^Growing nodule, high-risk history, before surgery or local therapies.

^*c*^In accordance with the presence of 1 or more suspicious findings.

^*d*^FNAB is recommended for subcapsular or paratracheal nodules and those associated with suspicious clinical findings (e.g., dysphonia); suspicious lymph nodes or extrathyroidal spread; a positive personal or family history of thyroid cancer; or a personal history of head and neck irradiation.

Within each risk class, size cutoffs are used to identify lesions whose FNAB can safely be deferred. Avoiding unnecessary FNABs is an important goal. Aside from cost considerations, these procedures can be associated with complications, albeit minor and transient (e.g., mild bruising, soreness, swelling, neck discomfort) (16). More important, inconclusive cytology results are by no means rare, and they often lead to additional testing (frequently quite expensive) and/or diagnostic surgery, undertaken for the purpose of confirming that the nodule is indeed benign. The accuracy of the risk estimates generated by the systems shown in Table 1 has in some cases been validated in retrospective (17-20) and/or prospective studies (21-23), and their performance has also been confirmed by a recent meta-analysis (24). These classifications also guide the timing of subsequent long-term follow-up evaluations and the eligibility of suspicious nodules for management limited to active surveillance.

As shown in Table 1, the nodule aspects considered in in the risk-estimation process are fundamentally the same with all these systems—structural composition, echogenicity, shape, margins, and echogenic foci—and risk classes are defined by sets or clusters of 2 or more nodule features (11). The exception to the latter rule is the American College of Radiology (ACR) TIRADS. With this system, the same key aspects of the nodule are assessed individually. Each is rated with a numerical score, and the sum of the 5 scores determines the risk class to which the nodule is assigned.

There is some degree of heterogeneity across systems in terms of the definitions of certain nodule features, the relative weight assigned to individual features, and the size criteria used for FNAB recommendations. Substantial variability has also been observed in the different systems’ ability to decrease the number of unnecessary FNABs. In 1 recent prospective analysis (21), the ACR TIRADS outperformed the 4 other widely used systems tested in reducing the number of biopsies performed on nodules ultimately diagnosed as benign: more than one-half of the biopsies would have been classified by the ACR system as deferrable, with a false-negative rate of only 2.2%. Although developed mainly for detection of papillary thyroid cancers (PTCs), the sonographic risk-stratification systems also seem to provide reliable recommendations for FNAB of follicular thyroid cancers (25), medullary thyroid cancers (26), and metastases to the thyroid gland (27).

Most physicians in the United States (28), Spain (29), and Italy (30) report using TIRADS or similar classifications when performing thyroid ultrasonography. However, most US reports in routine practice provide insufficient data for risk stratification (31). The optimal effectiveness of these systems in the real-world clinical practice also depends on the adoption of uniform terminology and accurate, nonambiguous definitions of the features being assessed. For these reasons, the European Thyroid Association (14), the Korean Society of Thyroid Radiology (15), and the ACR (32) have developed specific lexicons to be used with their risk-assessment systems. The definitions of certain critical nodule descriptors, such as echogenicity (33), shape (34), hyperechoic foci (35), and extrathyroidal extension (36), can significantly impact the diagnostic performance of thyroid US. As a result, interobserver agreement for US-based risk-stratification systems remains only fair to moderate (kappa, 0.34-0.44) (10). Specific training involving joint evaluation of images can be useful to increase operators’ ability to recognize these features, thereby improving the reproducibility for all classifications, even among trained clinicians with similar levels of experience (37, 38). To address this problem, the International Thyroid Nodule Ultrasound Working Group, a multidisciplinary alliance of physicians with expertise in thyroid nodule sonography, is attempting to devise a unified, international set of guidelines that is based on a validated lexicon and incorporates state-of-the-art techniques and research data (39). Standard B-mode US examinations can also be expanded to include elastographic (strain or shear-wave techniques) analysis (40) of the stiffness of the nodular tissue and contrast-enhanced assessments of its perfusion and vascularity (41). Both approaches have produced promising results and their use for US-based risk-stratification has been proposed (40, 41). Thus far, however, adoption of these proposals has been limited owing to availability and reproducibility issues. Other novel approaches include the use of software applications capable of performing automated analysis for extracting quantitative parameters from US images. These tools may be the basis for computer-aided diagnosis systems that can generate an automated “second opinion.” Some findings suggest that machine learning approaches are as accurate (42) or even more accurate (43) than expert radiologists in discriminating between malignant and benign thyroid nodules.

## Clinical risk factors

The prevalence of thyroid nodules increases with age, and most are detected in individuals older than 40 years of age. In addition to the sonographic appearance of the nodule, other factors have to be considered when deciding whether or not FNAB should be performed. Some are thought to be predictive of nodule development or malignancy (e.g., serum levels of TSH, autoantibodies, obesity), but the evidence for these associations is currently inconclusive. TSH should nonetheless be measured in all patients to rule out the possibility of a hyperfunctioning nodule. The latter lesions do not require biopsy because they are virtually always benign.

Recognized risk factors for thyroid malignancy are medical irradiation during childhood (44), accidental exposure to ionizing radiation from fallout in childhood or adolescence (45, 46), a family history of thyroid cancer, or hereditary syndromes that include a predisposition to thyroid cancer (e.g., PTEN hamartoma tumor syndrome, Carney complex, Werner syndrome) (13). Nodules that are firm, fixed, or rapidly growing require prompt evaluation (47). Recently, the intraglandular location of the nodule has also been confirmed to be an independent risk factor for malignancy. Nodules arising in the isthmus are the most likely to be diagnosed as cancer, whereas those found in the lower third of a lobe carry the lowest risk (48), as compared with those in the middle (49) or upper pole of the lobe (50).

These factors are typically not considered in risk stratification algorithms, but may influence the course of action in shared decision-making with patients (39).

## Cytology and molecular testing

Fine-needle aspiration cytology is the next step in the triage of a thyroid nodule. It should be reserved for lesions found to be sufficiently suspicious on the basis of US and clinical findings. The results play a key role in optimizing subsequent management. The Bethesda System for Reporting Thyroid Cytopathology (BSRTC) was discussed in 2007 by a panel of experts at the U.S. National Institutes of Health in Bethesda, MD. The first edition of the system was published in 2010, and an updated version followed in 2018 (51). The BSRTC is widely used in the United States, and it has served as a model for similar tiered classification schemes developed more recently in other parts of the world (52).

The robust diagnostic framework provided by the BSRTC offers valuable guidance in developing management strategies for patients with thyroid nodules (53). Nonetheless, several potential diagnostic pitfalls exist that can lead to false-positive, false-negative, nondiagnostic, or indeterminate results (54). Cytology itself has limitations: it cannot, for example, distinguish between follicular-patterned hyperplastic/adenomatoid nodules, follicular adenomas, follicular carcinoma, and some cases of follicular variants of papillary thyroid carcinoma. Thyroid cytology can be considered only a screening test for these follicular-patterned lesions, the results of which will almost invariably reported as “indeterminate,” that is, assigned to Bethesda class III (“atypia of undetermined significance” or “follicular lesions of undetermined significance”) or IV (“follicular neoplasm or suspicious for a follicular [or Hürthle cell] neoplasm”). For most papillary thyroid cancers, as well as medullary, poorly differentiated, and undifferentiated carcinomas, the cytology report will usually be unambiguously diagnostic (Bethesda class VI, malignant), whereas some degree of uncertainty persists for nodules assigned to Bethesda class V (suspicious for malignancy) nodules, which is associated with a very broad range of malignancy risks (53).

## What to do with indeterminate results?

As noted, the term “indeterminate cytology” refers to Bethesda class III or class IV findings, which are associated with expected malignancy rates of 10% to 30% and 25% to 40%, respectively. The options suggested for identifying these nodules includes repeat FNAB for cytology and/or molecular testing and diagnostic lobectomy. Some data suggest that a repeat US risk stratification can be useful in predicting malignancy and in planning further steps for the management of indeterminate nodules (55-58), or at least those in Bethesda class III (59, 60). However, if nodules are properly selected beforehand, and the pretest risk of malignancy is high, the utility of this approach may be reduced (61, 62).

Cytological assessment of a second fine needle aspirate is commonly used, but it provides a definitive diagnosis for only 40% of class I (nondiagnostic) and III nodules (63). If the second cytological study is still indeterminate, diagnostic surgery (usually lobectomy) has traditionally been the only route to a definitive pathological diagnosis. It is obviously expensive and associated with some risks. And if the nodule proves to be malignant, reoperation (completion thyroidectomy) is often indicated, with added risks and costs. Up to 60% of patients undergoing lobectomy for an indeterminate nodule are likely to be over- or undertreated at initial surgery (64).

Molecular testing of the FNAB samples is a newer approach that can reduce the need for diagnostic surgery. The tests developed for this purpose over the past 10 years are based on 3 main molecular approaches: testing for somatic mutations, gene expression evaluation, and microRNA (miRNA)-based classifiers (65-71). The current version of the ThyroSeq test (version 3) involves targeted next-generation sequencing analysis of 112 cancer-related genes for point mutations, gene fusions, copy number alterations, or abnormal gene expression. When validated on 257 cytologically indeterminate nodules with surgical pathology reports, it displayed good sensitivity (94%) and specificity (82%), with a negative predictive value of 97% and a positive predictive value of 66%. The authors concluded the test might eliminate the need for diagnostic surgery in up to 61% of patients with indeterminate nodules (66). The Afirma test was originally based on microarray analysis of mRNA expression profiles. The current version, the Afirma Genomic Sequencing Classifier (GSC), includes 12 classifiers composed of 10,196 genes (RNA sequencing approach). Compared with the previous version, the new test correctly classifies more indeterminate nodules as benign and displays improved specificity (68.3%) and positive predictive value (47.1%), with a sensitivity of 91.1% and a negative predictive value of 96.1% (67). It is expected to further reduce the frequency of diagnostic surgery, based on the results of independent studies (72-74). In short, the ThyroSeq and Afirma assays currently have positive and negative predictive values that make them suitable for use in both rule-in and rule-out testing. ThyroPrint, a gene-expression classifier based on interrogation of only 10 genes has also displayed good performance (sensitivity 96%, specificity 87%, and positive and negative predictive values of 78% and 98%, respectively) in an internal, multicenter validation study (70).

A well-known limitation of mutation-based approaches is related to the occurrence of *RAS* mutations in a wide variety of thyroid tumors, including follicular adenoma, noninvasive follicular thyroid neoplasm with papillary-like nuclear features, encapsulated and unencapsulated follicular-variant PTC, classic PTC, medullary thyroid cancer, poorly differentiated thyroid cancer, and anaplastic thyroid cancer. When clonally present, mutant *RAS* is an oncogene, and nodules harboring these mutations should be considered neoplastic. However, recent findings show that the presence of a RAS mutation alone is not a helpful marker of malignancy. The few cancers with this mutation prove to be low-risk tumors with fairly indolent behavior (75, 76).

Other available approaches are based on expression levels of miRNAs, small, highly conserved, noncoding RNA molecules capable of influencing the expression of messenger RNAs and impacting multiple pathways (77). Differential miRNA expression has been described in distinct thyroid cancer subtypes and is also linked to the differentiation or progression status of these tumors (78). MiRNAs have also been proposed as circulating biomarkers of thyroid cancer in peripheral blood (79). As a result, miRNA gene expression classification has been proposed as a complementary molecular test that can further improve predictive values and refine surgical management (69, 71). Promising results have been reported for a combination assay that includes miRNA classification (ThyraMIR) and next-generation sequencing mutation analysis (ThyGeNEXT) (positive predictive value 74%, negative predictive value 94% (68)), but the assay has yet to be subjected to independent validation. Molecular tests require a dedicated needle pass, the collection of residual material in the needle hub, liquid cytology remnants, or the recovery of cells from routinely prepared cytology slides. In some cases, a repeat dedicated biopsy is needed; in others (i.e., ThyroSeq, ThyGeNEXT/ThyraMIR, and ThyroPrint) can also be performed using the original cytology slide.

Meaningful comparison of these tests in terms of their diagnostic performance is extremely difficult for several reasons. The currently available data come from studies that differ significantly from one another in cohort selection criteria, sample sizes, malignancy rates, study designs, and applied reference standards (e.g., not all lesions are surgically resected, the issue of inter-pathologist variability is not always addressed). In addition, no attempts have been made to conduct direct head-to-head comparisons, using more than 1 test on the same samples. Furthermore, the high costs of these tests limit their use in many countries. However, when hypothetical modeling was used to compare surgery versus molecular testing for the management of indeterminate nodules, both of the major molecular approaches discussed previously proved to be considerably more cost-effective than diagnostic lobectomy, and the Thyroseq v. 3 was more cost-effective than the Afirma GSC (80). If, on the basis of all clinical, imaging, and cytologic findings, the sole aim of surgery is diagnostic, molecular testing should definitely be considered.

## Follow-up examinations: what to look for

**Figure 1.**
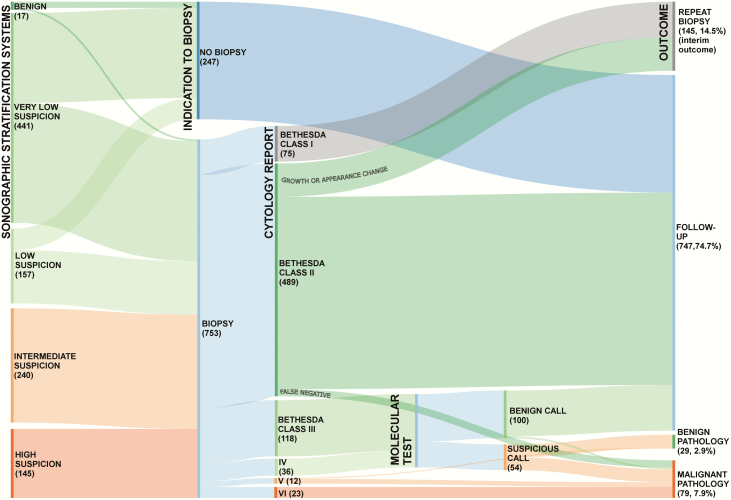
Alluvial flow diagram showing simulated management and outcomes for 1000 newly discovered thyroid nodules. The distributions of ultrasound (US)-defined risk classes, US-defined fine-needle aspiration biopsy (FNAB) indications, and Bethesda cytology class were derived from published findings (21). US risk-stratification is that recommended by the American Thyroid Association (ATA) Guidelines. Nodules not classifiable with the ATA system are included in the intermediate-suspicion category. Nondiagnostic nodules with very-low-suspicion or low-suspicion US findings can be managed with US surveillance, but repeat FNAB is indicated for those with intermediate- or high-suspicion US findings (81): in this diagram, all are shown as undergoing repeat biopsy. Bethesda II nodules require repeat biopsy only if the US-based risk class increases during surveillance (frequency: ~15% over 5 years of follow-up) (82). The false-negative rate is less than 3% (e.g., sampling error; for high-suspicion nodules with Bethesda II cytology, repeat biopsy is suggested) (83). For illustration purposes, all indeterminate nodules are shown as undergoing molecular testing (regardless of other possible options). The hypothetical molecular testing approach depicted has a benign call rate of 65%, a positive predictive value of 50%, and a negative predictive value of 96% (72-74). For high-suspicion nodules classified as benign by molecular testing, repeat biopsy is indicated. All Bethesda V and VI nodules are referred for surgery. Expected malignancy rates are 80% and 99%, respectively.

Fig. 1 shows the simulated management strategies and outcomes of 1000 newly discovered thyroid nodules (21, 72-74, 81-83). The overall management pathway is based on the US-risk stratification of the target lesions and the cytology assessment (if any). These scenarios do not include symptomatic thyroid nodules that are already candidates for resection regardless of their sonographic features. In these cases, a biopsy might be performed to clarify the best surgical approach, but the results would not change the indication for surgery itself (84). The distributions of US-defined risk classes, US-defined FNAB indications, and Bethesda cytology class were derived from published findings (21). For illustration purposes, all indeterminate nodules are shown as undergoing molecular testing, although other options are offered. However, if these alternative approaches, guided by clinical and US data, had been used, it is unlikely that the final number of resected nodules and their malignancy rate would be significantly different from those shown in Fig. 1. The hypothetical molecular testing approach depicted has a benign call rate of 65%, a positive predictive value of 50%, and a negative predictive value of 96% (72-74).

After the initial diagnostic workup described, very few of the nodules (10.8%) will be subjected to surgery, but a high percentage of those that are resected will prove be malignant (73.1% of the resected nodules; 7.9% of all nodules). Some nodules (14.5%) will require a repeat biopsy, immediately or during the long-term follow-up, to be classified. For Bethesda II nodules, a second cytological assessment is indicated only if the US-based risk class increases during surveillance (frequency: ~15% over 5 years of follow-up) (82). Three-quarters of all nodules (74.7%) will be classified as benign and managed with long-term sonographic surveillance. False negatives (usually the result of sampling errors) are uncommon (less than 3%), but repeat biopsy should be considered for high-suspicion nodules with Bethesda II cytology (83). Most of these nodules (≈85%) will remain asymptomatic with no signs of growth and will therefore not require any treatment. This estimate is based on findings from a 5-year prospective study of 1567 benign thyroid nodules (85), which have subsequently been confirmed by retrospective studies (86).

**Figure 2.**
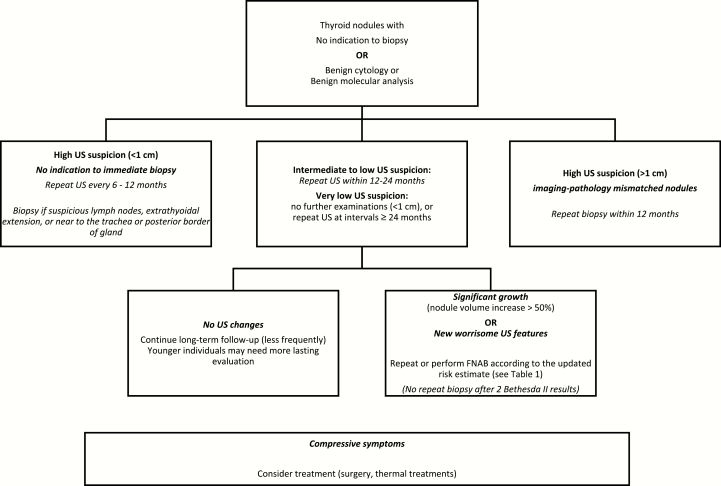
Suggested management and follow-up of nodules with no indication for immediate biopsy and those cytologically classified as benign.

The aim of long-term surveillance should be to detect any previously missed malignancy and monitor thyroid nodule growth. The sonographically estimated malignancy risk also provides guidance in planning effective follow-up (Fig. 2). The algorithm shown in the figure is based on literature data, practice guidelines, and the authors’ own experience. Thyroid FNAB cytology has a very low false-negative rate (<3%); similar findings have been reported for molecular analysis of FNAB fluid (Table 2). However, nodules with highly suspicious features on the initial US examination—even if they have been cytologically or molecularly classified as benign—warrant a repeat biopsy within 12 months. Repeat biopsy is also indicated if new suspicious features or significant growth is observed during follow-up. In a subgroup analysis of a prospective cohort, the US-estimated malignancy risk class of 13.2% to 29.4% of the nodules increased during the first 5 years of follow-up. However, the risk-class increase was associated with a recommendation for FNAB for only 6% to 8% of the nodules whose biopsies had been deferred at baseline (82), and none of the increased risk estimates was associated with discovery of a new malignancy. The development of new nodules is quite common during surveillance, but only 3% to 7% of these lesions meet the criteria for biopsy. Some authors maintain that a sonographically documented change in the appearance of a nodule classified as benign (particularly its margins) is the most reliable indication for repeat FNAB (87). The growth alone of a such nodules emerged from a meta-analysis as a relatively poor predictor of malignancy (88). More recently, however, a prospective study found that nodules displaying significant growth during follow-up (diameter increases exceeding 2 mm per year) are significantly more likely to be malignant than slower growing nodules (relative risk, 2.5; 95% confidence interval, 1.6–3.1; *P* < 0.001) and therefore warrant repeat biopsy (89). Growth is also a concern because it can cause compressive symptoms. Size increases are more likely in younger individuals and patients with multiple or large nodules (85).

**Table 2.** Minimally Invasive Techniques for Treatment of Symptomatic, Benign Thyroid Nodules

Method	Mechanisms	Candidate Nodules	Expected Volume Reduction	Adverse Effects	Cost Estimate
Percutaneous ethanol injection (PEI)	Dehydration of cytoplasmic proteins, coagulation necrosis, and fibrosis	Cystic or predominantly cystic nodules	~60%	• Pain • Burning sensation • Hematoma • Dyspnea • Voice change	Simplest, least expensive option ($50-$100)
Radiofrequency ablation (RFA)^*a*^ (101, 103, 104)	Thermal coagulation necrosis	Mixed or solid nodules	47.7%-96.9%	Overall complication rate 2.11% (major complications 1.27%) **Major:** • Voice change • Nodule rupture • Hypothyroidism • Brachial plexus injury **Minor:** • Pain • Thermal propagation outside of the thyroid • Fever • Skin burns • Hematoma • Transient hyperthyroidism/transient thyroiditis	Equipment $25,000; $800 per session
Laser ablation (LA)^*a*^ (101, 102)	Thermal coagulation necrosis	Mixed or solid, functional, or nonfunctional nodules	62 ± 22%^*b*^	• Pain (10%) • Fever (8%) • Vasovagal reaction (1%) • Voice change (0.5%) • Hematoma (0.4%) • Skin burn (0.1%)	Equipment with built-in laser source: $12,000 Nd:YAG laser source: $15,000-$20,000 $500 per session
Microwave ablation (MWA) (105-107)	Thermal coagulation necrosis	Mixed or solid nodules	50%-70 % depending on nodule composition (solid require more energy than cystic nodules)	• Pain (25%) • Transient voice change (1%) • Hematomas • Burns (2/30) • Horner syndrome (1/30)	Equipment: $35,000 $400 per session
High-intensity focused ultrasound (HIFU) (98-100)	Thermal coagulation necrosis	Mixed or solid nodules	49%-69%	• Hypothyroidism (1.4%-2.3%) • Hoarseness • Neck swelling	Focused thermal tissue destruction without needles; Equipment: $400,000 $350 per session

^*a*^Laser fibers deliver energy to the target more accurately than radiofrequency electrodes. The efficacies of the 2 techniques are potentially similar in the hands of operators with the same levels of skill and experience. RFA appears to be superior for benign solid nodules (108); LA seems slightly more effective for nodules > 30 mL (101).

^*b*^The rate of decrease depends on nodule type, vascularity, energy used, operator experience (109).

On the whole, the recent findings and increasing use of comprehensive US-based risk stratification systems should reduce the “diagnostic burden” related to nodules classified as benign: they can be safely followed with visits every 2 or 3 years, and the frequency can be even further reduced if no changes have been noted at previous visits.

Patients whose nodules are cytologically classified as malignant or suspicious for malignancy (Bethesda classes V and VI; or suspicious molecular analysis) should generally be referred for surgery. However, for those with subcentimeter, intrathyroidal cancers with no high-risk features, active US surveillance can be proposed in lieu of immediate surgery (3, 4). The first study published on this issue in 2003 found that more than 70% of microPTCs remained stable during surveillance, and the frequency of spread to the locoregional lymph nodes was quite low (~1%) (90). The active surveillance strategy does not merely delay surgery: the likelihood of disease progression diminishes with age, and older patients are therefore less likely to require surgery during their lifespan (91). Similar results have been reported worldwide (92, 93).

Active surveillance protocols initially provide for US neck examinations every 6 months. Once disease stability has been documented—in general, with 2 years of serial US examinations showing no evidence of progression—the examination frequency can be reduced to every 1 to 2 years or more (94). In contrast, if surveillance does reveal evidence of progression (i.e., an increase ≥3 mm in the maximum diameter of the nodule, growth toward the dorsal surface of the gland or toward nearby structures, or the appearance of lymph node metastases) (95, 96), surgery is indicated. These data provide a solid background for recommendations to avoid immediate biopsy and adopt an US-based surveillance strategy for subcentimeter, intrathyroidal nodules even if sonographically suspicious (13).

## Management

### Benign nodules

Benign thyroid nodules requiring treatment are rare. The most common are hyperfunctioning nodules and nodules whose growth is associated with compression of vital structures like the trachea or esophagus, general neck discomfort, and/or cosmetic problems—all of which can negatively impact quality of life. Surgery is an option in these cases, but there are also several nonsurgical, minimally invasive alternatives. These include US-guided ablation procedures involving percutaneous ethanol injection (the traditional method and currently the least expensive) or the application of heat in the form of laser, radiofrequency, high-intensity focused US, or microwave energy. Radiofrequency and laser ablations can significantly reduce nodule volumes (97). As shown in Table 2 (98-109), these techniques differ in terms of their indications, adverse effects, and associated costs. Hyperfunctioning nodules can also be treated with radioiodine.

High-intensity focused US is a newer needle-free technique that is producing promising results (98-100), but it requires further clinical validation. More evidence and experience are also needed before microwave ablation is used on a large-scale basis. The use of these techniques for the treatment for symptomatic benign nodules has been addressed by several groups of experts (110-113). In general, consensus statements by these groups list US-guided aspiration as the first-line treatment for cystic or predominantly cystic nodules. Ethanol injection can be used for recurrences, and thermal ablation techniques are reserved for cases in which symptoms persist after ethanol. Thermal ablation can be used for nodules that are predominantly solid and/or growing, but only after the benign nature of the lesion has been confirmed with 2 serial FNABs and serum calcitonin assessment. For nodules with lower risk features on US or autonomously functioning lesions, a single aspirate with benign cytology is sufficient (110, 111). The clinical and US-based follow-up of benign nodules that undergo treatment require expert clinical and US evaluation, because the morphologic features may change over time. If regrowth occurs, a new cytological assessment is indicated.

When surgery is indicated, decisions on the extent of resection will depend on multiple factors, including symptoms, the presence of contralateral nodules, thyroid functional status, comorbidities, family history, surgical risk, and patient preferences (84). Common reasons for surgery are large goiters, local compressive symptoms or progressive nodule or thyroid enlargement, or large toxic multinodular goiters. In most patients with multiple nodules, both lobes of the gland are involved and total thyroidectomy is necessary. Consensus is lacking on the procedure of choice for patients with an asymmetric nodular goiter. In some cases, lobectomy can be considered as a safer alternative to total thyroidectomy. However, it requires long-term follow-up, is associated with nodule recurrence risk (114), and may subsequently require a second operation (115).

### Indeterminate and suspicious nodules

For cytologically indeterminate nodules that cannot be molecularly classified as benign, lobectomy with isthmusectomy is generally the procedure of choice. However, thyroidectomy may be indicated in patients with larger indeterminate nodules (≥3-4 cm), nodules displaying progressive growth and/or worrisome features on ultrasound, or a family history of thyroid cancer or radiation exposure (84). If preoperative molecular testing is not possible, seeking the opinion of a second pathologist may be worthwhile because thyroid cytology is characterized by substantial inter- and intraobserver variability, especially for nodules classified as indeterminate (116, 117).

Lobectomy and isthmusectomy (or rarely an isthmusectomy alone) is usually the least extensive procedure that can be considered when malignancy is suspected (84). (Cases eligible for active surveillance, as discussed previously, are the obvious exception.) In patients with 1- to 4-cm suspicious nodules, lobectomy or total thyroidectomy are both acceptable, whereas patients with large suspicious nodules, suspected extrathyroidal extension, or suspected metastases (locoregional or distant) should undergo total thyroidectomy.

Lobectomy offers several advantages over total thyroidectomy. It virtually eliminates the risks of permanent hypoparathyroidism and bilateral recurrent laryngeal nerve injury and substantially reduces the rates of permanent unilateral recurrent laryngeal nerve palsy (0.6% versus 1.3%) (118). Furthermore, 50% to 80% of the patients who undergo lobectomy do not require thyroid hormone replacement therapy (the likelihood varies according to the preoperative TSH level and the presence of thyroid autoimmunity.) (114, 115). Minimally invasive US-guided ablation techniques are also being proposed by some groups for nonsurgical treatment of small suspicious nodules (119, 120).

## Conclusions

The evaluation and management of patients with thyroid nodules is no longer a 1-size-fits-all proposition. The tailored approach advocated today requires a careful assessment of each nodule to determine the likelihood that it is malignant and the chances that it will cause symptoms. Very few nodules will require an intensive workup that includes cytology and molecular testing of FNAB samples: for the vast majority, a systematic cervical US examination with assessment of clinical risk factors will provide a reliable foundation for identifying the initial management strategy. After an appropriate initial assessment, the frequency of subsequent surveillance visits for most nodules can be safely reduced compared with currently used schedules. This is particularly relevant for frail, elderly individuals, as they are unlikely to be harmed by the thyroid tumor itself, and overmedicalization can cause more harm than good. In these populations, surveillance can safely be confined to a periodic clinical examination. If surgery is needed, resections can often be less extensive. In some cases, minimally invasive, percutaneous approaches are a viable alternative to surgery. When there are multiple options, they should be discussed as frankly as possible with the patient, outlining the advantages, limitations, benefits, and risks of each. The goal is to identify the best strategy for the individual patient, in terms of disease outcomes and quality of life, avoiding the pitfalls of overdiagnosis and overtreatment. For health professionals, this means learning to work with some degree of clinical uncertainty rather than automatically resorting to intensive testing and intervention, and by weighing patients’ expectations and demands.

## Abbreviations

ACR: American College of Radiology
BSRTC: Bethesda System for Reporting Thyroid Cytopathology
FNAB: fine-needle aspiration biopsy
GSC: Genomic Sequencing Classifier
PTC: papillary thyroid cancer
TIRADS: Thyroid Imaging Reporting and Data System
US: ultrasonography

## Additional Information

***Disclosure Summary*:** The authors have no conflicts of interest to disclose.

***Data Availability:*** Data sharing is not applicable to this article as no datasets were generated or analyzed during the current study.
